# CXCL4 suppresses tolerogenic immune signature of monocyte‐derived dendritic cells

**DOI:** 10.1002/eji.201948341

**Published:** 2020-07-10

**Authors:** Sandra C. Silva‐Cardoso, Weiyang Tao, Beatriz Malvar Fernández, Marianne Boes, Timothy R.D.J. Radstake, Aridaman Pandit

**Affiliations:** ^1^ Center for Translational Immunology University Medical Center Utrecht Utrecht The Netherlands; ^2^ Department of Rheumatology & Clinical Immunology University Medical Center Utrecht Utrecht The Netherlands; ^3^ Department of Pediatrics University Medical Center Utrecht Utrecht The Netherlands

**Keywords:** C1q, CXCL4, DNA methylation, Monocyte‐derived dendritic cells, RNA sequencing

## Abstract

RNA sequencing and DNA methylomic profiling were performed after differentiating monocytes for 6 days into moDCs with/without CXCL4 presence. We show that CXCL4 downregulates genes associated with tolerogenicity in DCs including C1Q. Expression profiles of C1Q genes were negatively correlated with their DNA methylation profiles and with immunogenic genes.

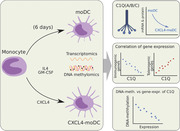

**Abbreviations**
HV: healthy volunteer
moDC: monocyte‐derived dendritic cells

DCs are sentinels that play a crucial role in inducing peripheral tolerance and immune response. Molecules resulting from cell damage, microbial products, and cytokines modulate DCs toward tolerogenic or immunogenic responses. Such alterations of DC function can contribute toward autoimmunity.

CXCL4 is a chemokine that is known to modulate DC functions and has been implicated in cancer and autoimmunity. Previously, we showed that differentiation of monocyte‐derived DCs (moDCs) in the presence of CXCL4 promotes the expression of activation molecules, sensitivity toward TLR‐mediated stimulation, and potentiated activation of inflammatory responses by T‐cells. The control of tolerogenic and immunogenic responses by DCs involves intensive transcriptional and epigenetic reprograming, including DNA methylation. Thus, here we investigated the effect of CXCL4 on markers of DC immunogenicity and tolerogenicity using RNA sequencing and DNA methylomics.

Modulating the function of DCs is an active area in immunological and translational research. Several protocols have been proposed for the generation of tolerogenic and immunogenic DCs resulting in establishment of multiple candidate biomarkers that characterizes their function [1, 2].

Previously, we showed that exposure to CXCL4 drives moDCs to a semi‐mature phenotype and function [3]. Here, we cultured monocytes from five healthy volunteers (HV) with IL‐4 and GM‐CSF to differentiate into moDCs or with IL‐4, GM‐CSF, and CXCL4 to differentiate into moDCs (CXCL4‐moDCs). We performed RNA sequencing analysis on day 6 cultured moDCs and CXCL4‐moDCs (see Supporting Information for details). We observed that genes associated with immunogenic DC responses such as *CD86*, *CD83*, *HLA‐A*, *CCR7*, *CCL17*, *FSCN1*, *LAMP3*, *SOD2*, *CD40*, and *ICAM1* were upregulated on CXCL4‐moDCs. Interestingly, the expression of CSF 1 receptor (*CSF1R*), which is downregulated in inflammatory DCs [4], was downregulated on CXCL4‐moDCs (Fig. 1A, Supporting Information Fig. S1A). *CD80*, a co‐stimulatory molecule associated with response to stimuli, was not differentially expressed (Supporting Information Fig. S1A). Moreover, genes associated with DC tolerogenicity such as *IL10*, *SLAMF1*, *SMAD3*, *FZD2*, *F13A1*, *STAB1*, *CTSC*, *FCGR2B*, *CD37*, and *GILZ* (*TSC22D3*) were downregulated by CXCL4 (Fig. 1A, Supporting Information Fig. 1B) [5].

**Figure 1:**
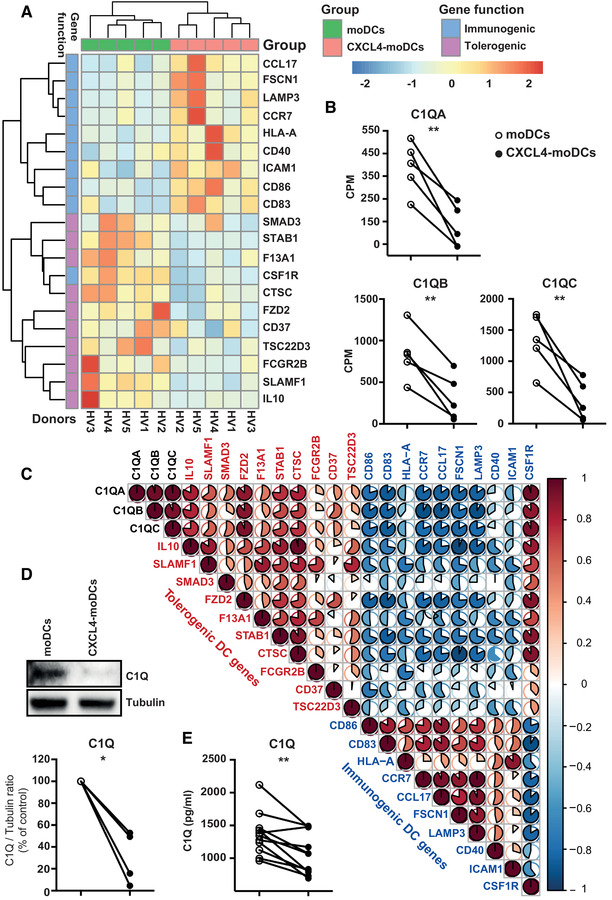
CXCL4 drives dramatic up‐regulation of immunogenic signature and down‐regulation of tolerogenic markers, inclusively C1q. (A) Heat map showing immunogenic and tolerogenic gene signatures. The colour scheme represents gene expression and is shown as Z‐scores. (B) Expression of *C1QA*, *C1QB*, and *C1QC* genes on moDCs and CXCL4‐moDCs. (*n* = 5 Healthy Volunteer or HV). Likelihood ratio test. ^**^ *P* < 0.01. (C) Pearson correlation analysis between the expression of *C1QA*, *C1QB*, and *C1QC* genes (black text) and tolerogenic (red text) or immunogenic (blue text) signature genes. Color scheme gradient and pie graphs represent the correlation coefficients between comparisons. Data shown for 5 HV, all from independent experiments. (D) Western blot analysis of C1q and tubulin. Representative blot of 4 HV is shown (4 independent experiments). Below, we show the quantification for 4 HV. Paired *t*‐test. ^*^ *P *< 0.05; (E) Measurement of soluble C1q by Elisa (*n* = 11 HV, 8 independent experiments). Paired *t*‐test. ^**^ *P *< 0.005.

C1q has been shown to be critical for maintaining immune tolerance. We found that CXCL4 downregulates the expression of all three C1q genes (*C1QA*, *C1QB*, and *C1QC*) (Fig. 1B and D). C1q genes negatively correlated with immunogenic genes (Fig. 1C), and positively correlated with the other tolerogenic genes. In fact, among all tolerogenic genes, *C1QA*, *C1QB*, and *C1QC* genes exhibited the strongest negative correlation with most of the immunogenic markers. Additional validation showed that CXCL4 diminished C1q protein expression (Fig. 1D) and CXCL4‐moDCs released lower amounts of C1q in comparison to moDCs (Fig. 1E).

Changes in DNA methylation have been associated with aberrant gene expression and autoimmune disorders [6]. CXCL4 induced hypermethylation in the promoter regions of all 3 C1q (*C1QA*, *C1QB*, *C1QC*) genes and in the gene body of *C1QB* and *C1QC* genes (Fig. 2A–C). All these hypermethylated regions were strongly negative correlated with the corresponding gene's expression (Fig. 2D–F). Additionally, we found that CXCL4 drives hypermethylation of individual CpGs across the *C1QA*, *C1QB*, and *C1QC* genes, and exhibits strong negative correlated with the corresponding gene's expression (Supporting Information Fig. S2A–C).

**Figure 2:**
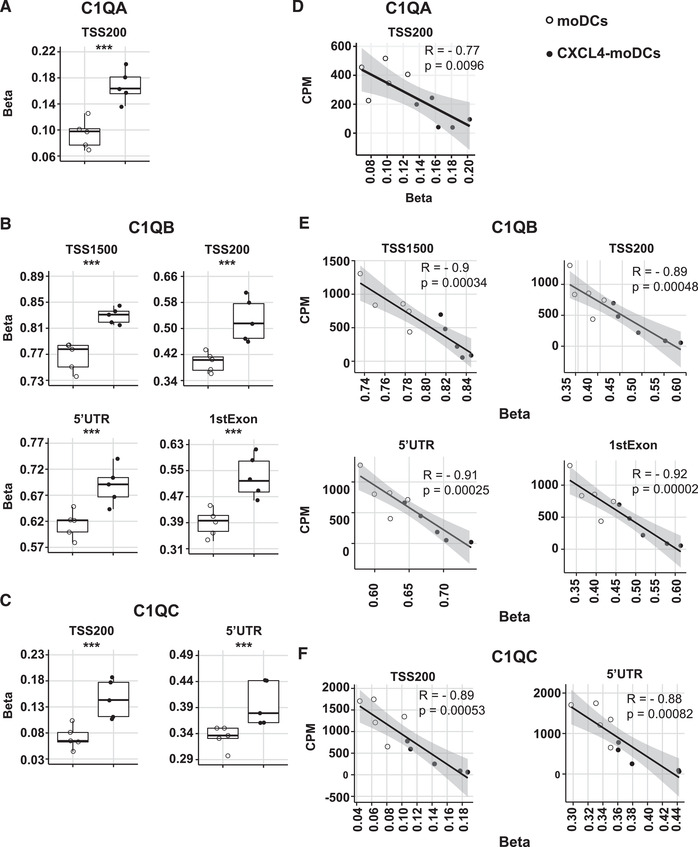
CXCL4 exposure during moDC differentiation associates with strong hypermethylation of C1q. DNA methylation analysis between moDCs and CXCL4‐moDCs of *C1QA* (A), *C1QB* (B), and *C1QC* (C) regions (1500 and 200 bp upstream of the transcription start site (TSS); 5’untranslated region (UTR), and first exon) (*n* = 5 HV). Likelihood ratio test. ^***^ *P *< 0.005. Correlation between differently methylated (D) *C1QA*, (E) *C1QB*, (F) *C1QC* regions and their corresponding gene expression, respectively. “R” represents Pearson correlation and “p” represents p‐value calculated by *t‐*test. Data shown for 5 HV, all from independent experiments.

Other genes associated with tolerogenic responses including *IL10*, *SLAMF1*, *STAB1*, and *CTSC* were hypermethylated in CXCL4‐moDCs and exhibited negative correlations between RNA expression and DNA methylation levels (Supporting Information Fig. S3A and B). However, it was not the same case for other tolerogenic genes (*F13A1*, *STAB1*, and *SMAD3*). Interestingly, no immunogenic gene exhibited significant negative correlation between RNA expression and DNA methylation levels (data not shown). Thus, CXCL4 mediates epigenetic modifications and transcriptional suppression of tolerogenic markers (especially C1q) to tip the balance between immunogenic and tolerogenic DCs.

Binding of C1q, the first component of the classical pathway of complement system, to PAMPs and apoptotic cell fragments results in the initiation of the complement system cascade and cell activation. C1q also functions as an opsonin that enables the detection and phagocytosis of PAMPs and apoptotic cell fragments either directly, or indirectly via binding to secreted antibodies and C‐reactive protein (CRP). Immature DCs and macrophages are able to secrete high levels of C1q in contrast to monocytes, mature DCs and T‐cells [7]. Primary C1q deficiency in humans [8] and C1q KO mice [9] have been shown to result in autoimmune conditions such as systemic lupus erythematosus (SLE).

C1q has been consistently shown to be up‐regulated on tolerogenic DCs [1, 2, 10]. Inflammatory triggers were shown to diminish C1q production during DC maturation [7]. Here, we revealed for the first time that CXCL4 exposure epigenetically modified promotors of several tolerogenic markers, including C1q genes and repressed expression of C1q genes. Thus, CXCL4 suppresses tolerogenic DC phenotype and boosts immunogenic responses, and we elucidated C1q as a critical factor in CXCL4‐driven autoimmune diseases.

## Conflict of interest

The authors declare no financial or commercial conflict of interest.

## Supporting information

Supporting Information.
