# Supplementary material for: CXCL4 suppresses tolerogenic immune signature of monocyte‐derived dendritic cells
Source: Eur J Immunol. 2020 Jul 10;50(10):1598–601. doi: 10.1002/eji.201948341 (PMC7586983; doi:10.1002/eji.201948341)
Supplement: Supplementary file 1 — Supporting Information. [file EJI-50-1598-s001.pdf]

SUPPLEMENTARY INFORMATION

CXCL4 suppresses tolerogenic immune signature of monocyte-derived dendritic cells

Sandra C. Silva-Cardoso[1,2#]; Weiyang Tao[1,2#]; Beatriz Malvar Fernández[1,2]; Marianne Boes[1,3]; Timothy R.D.J. Radstake[1,2*]; Aridaman Pandit[1,2*]

Affiliations:

[1]Center for Translational Immunology, University Medical Center Utrecht, the Netherlands

[2]Department of Rheumatology & Clinical Immunology, University Medical Center Utrecht, the Netherlands

[3]Department of Pediatrics, University Medical Center Utrecht, the Netherlands

#,* Authors equally contributed

Full correspondence: Dr. A. Pandit (a.pandit@umcutrecht.nl) and Prof. Dr. T.R.D.J. Radstake (t.r.d.j.radstake@umcutrecht.nl)

Materials and methods

Generation of moDCs and preparation of RNA and DNA samples

Differentiation of moDCs was implemented as described before [22]. Briefly, blood was collected in accordance with institutional ethical approval, and peripheral blood mononuclear cells (PBMCs) were isolated by density-gradient centrifugation over Ficoll PaqueTM Plus (GE Healthcare). For the purification of monocytes we used anti-CD14 magnetic beads for positive isolation by an autoMACS Pro Separator-assisted cell sorting (Miltenyi Biotec). Monocytes were cultured for 6 days in RPMI 1640 with GlutaMAX (Life Technologies), supplemented with 10% (v/v) heat-inactivated FCS (Biowest) and 1% (v/v) antibiotics (penicillin and streptomycin) (both from Life Technologies), and treated with recombinant human IL-4 (500 U/ml) and GM-CSF (800 U/ml) (both from R&D) on day 0 and 3 for moDC generation. For the generation of CXCL4-moDCs, recombinant human CXCL4 (10 μg/ml; PeproTech) was added on day 0 and day 3.

DNA and RNA were extracted from moDCs and CXCL4-moDCs on day 6 upon differentiation. Cells were lysed in RLTplus buffer (Qiagen) containing 1% (v/v) β-mercapto-ethanol (Sigma). DNA and RNA were purified using an Allprep Universal Kit (Qiagen) accordingly to manufacturer's instructions, and quantified using Qubit dsDNA HS Assay Kit and Qubit RNA HS Assay Kit, and measured using Qubit 2.0 fluorimeter (Invitrogen).

RNA sequencing and analysis

RNA-seq library was prepared using 100ng total RNA by the TruSeq kit (Illumina). The RNA-seq library was prepared using TruSeq kit (Illumina) and the library products were sequenced on an Illumina NextSeq 500 sequencer (25 million clean single-end reads of 75 bp) at Utrecht Sequencing Facility UMC Utrecht. Reads were aligned to Ensembl human genome (GrCh38, v79;

http://www.ensembl.org), using STAR aligner with the default parameters [29] and were counted using HTSeq package [30]. Likelihood ratio test (LRT) was performed to obtain differentially expressed genes using the DESeq2 (1.8.2) R/Bioconductor package, and genes with FDR adjusted p-value < 0.05 were considered to be differentially expressed. [31]. The raw read counts were normalized in each sample to count per million (CPM).

DNA methylation analysis

HumanMethylation850-BeadChip-based DNA methylation profiling (Illumina, Inc.) was performed according to the manufacturer's instructions at the GenomeScan (GenomeScan B.V., Leiden, The Netherlands). The CpG data obtained was quality checked and normalized using Beta-mixture quantile normalization (BMIQ) method in ChAMP (version 2.6.0) package [32]. The results of global integration of RNA-seq and DNA methylation are part of another manuscript (manuscript under review).

Cytokine quantification by ELISA

Secreted C1q was quantified by ELISA accordingly to the manufacturer's instructions (Human C1q ELISA Kit, Cat# E-EL-H0803, Elabscience) and measured on CLARIOstar micro-plate reader (BMG LABTECH) set to 450 nm.

Western blot

Cell lysates were resolved on 4-12% Bis-Tris SDS NuPAGE gels (Invitrogen). After blocking the membranes were probed overnight at 4°C with the Abs for C1q (PA5-29586, Invitrogen) and alpha-tubulin (Sigma, T9026). Membranes were extensively washed and incubated with secondary swine

anti-rabbit and goat anti-mouse HRP-conjugated Ab (Dako) for 1 hour at RT. The ratio between the levels of C1q and tubulin were calculated to determine the relative expression of C1q.

Statistical analysis

GraphPad Prism software (version 7) was used for statistical analysis of protein validation. Paired Student *t* test were used and significance was defined as *p*-value ≤ 0.05.

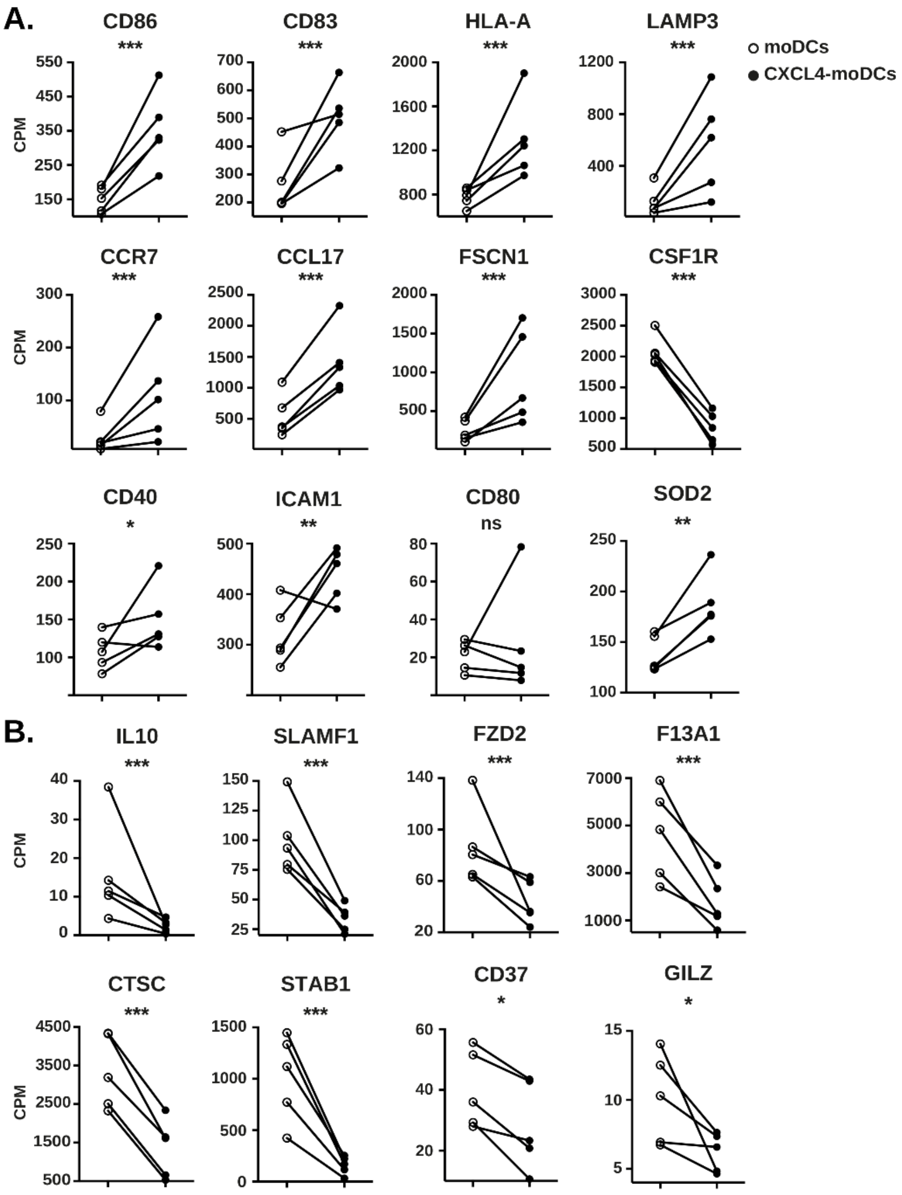

Figure S1. Exposure to CXCL4 during moDC differentiation leads to up-regulation of immunogenic and down-regulation of tolerogenic markers. RNA sequencing was performed on day 6 of differentiated moDCs and CXCL4-moDCs. Gene expression analysis of (A) immunogenic and (B) tolerogenic associated molecules in count per million (CPM). Lines connect samples of individual HV; Likelihood ratio test. $^{*}P<0.05$; $^{**}P<0.01$; $^{***}P<0.005$. Data shown for 5 HV, all from independent experiments.

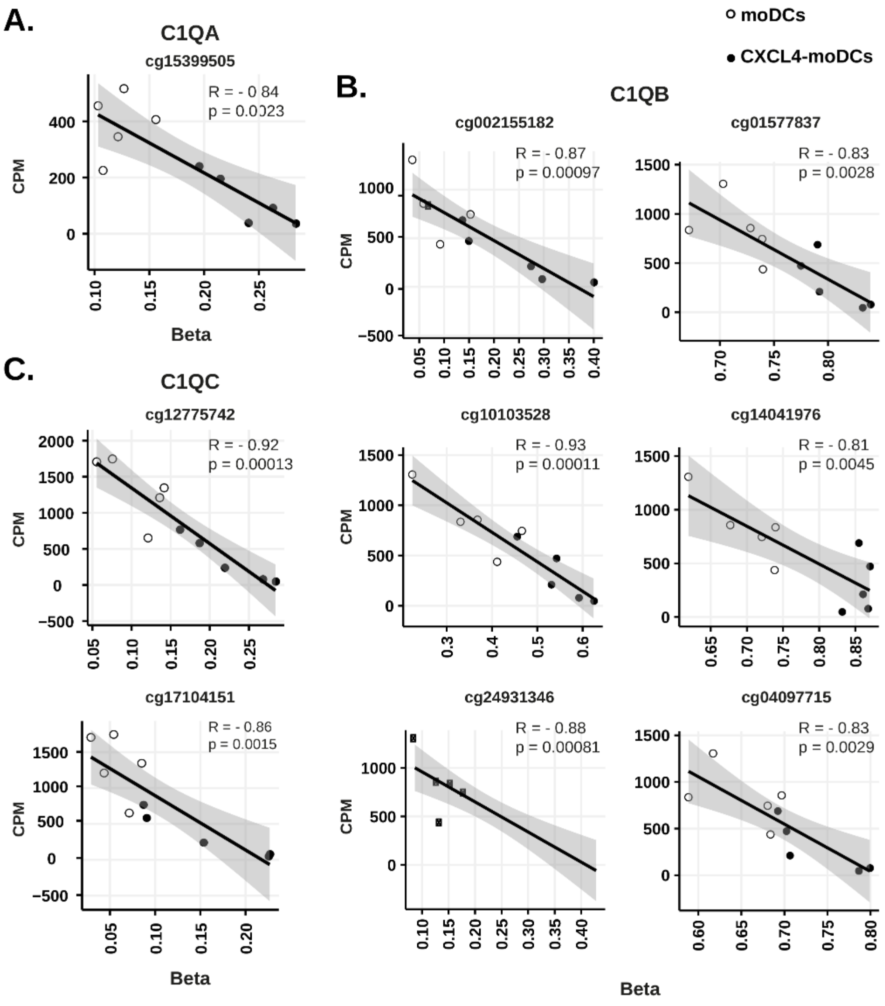

Figure S2. CXCL4 associates with strong hypermethylation of C1q CpGs. (A) Correlation between differently methylated *C1QA*, *C1QB*, *C1QC* CpGs and their corresponding gene expression, respectively**.** "R" represents Pearson correlation and "p" p value calculated by *t* test. Data shown for 5 HV, all from independent experiments.

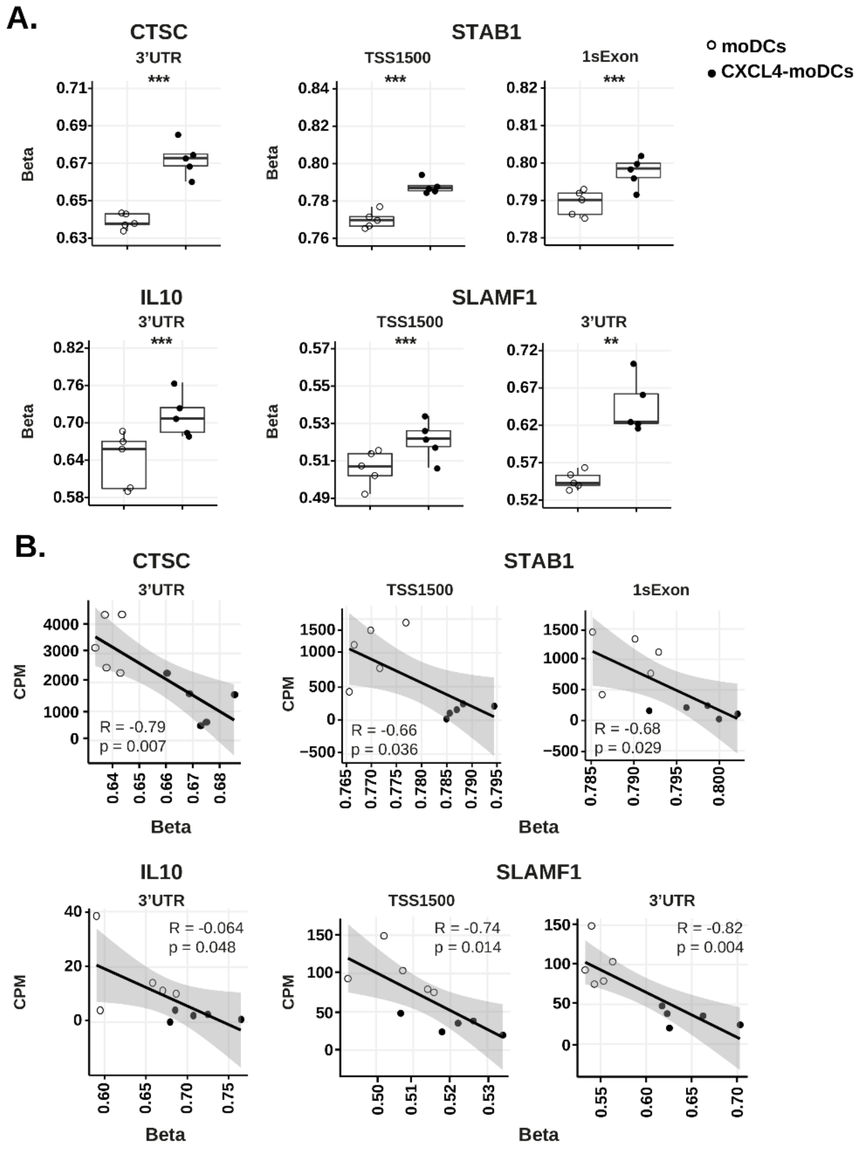

Figure S3. Down-regulation of particular tolerogenic markers by CXCL4 is associated with strong hypermethylation. (A) DNA methylation levels of *CTSC*, *STAB1*, *IL10* and *SLAMF1* between moDCs and CXCL4-moDCs. Likelihood ratio test. $^{**}P<0.01$; $^{***}P<0.005$. (B) Correlation between differently methylated regions (1500 upstream of the transcription start site (TSS); 1st exon and 3'UTR) and their corresponding expression for *CTSC*, *STAB1*, *IL10* and *SLAMF1* genes, respectively. "R" represents Pearson correlation and "p" p value calculated by *t* test. Data shown for 5 HV, all from independent experiments.
